# Singlet Triplet-Pair Production and Possible Singlet-Fission in Carotenoids

**DOI:** 10.1021/acs.jpclett.1c03812

**Published:** 2022-02-02

**Authors:** Dilhan Manawadu, Darren J. Valentine, Max Marcus, William Barford

**Affiliations:** † Department of Chemistry, Physical and Theoretical Chemistry Laboratory, University of Oxford, Oxford OX1 3QZ, United Kingdom; ○ Linacre College, University of Oxford, Oxford OX1 3JA, United Kingdom; ‡ Department of Chemistry, Physical and Theoretical Chemistry Laboratory, University of Oxford, Oxford OX1 3QZ, United Kingdom; ∇ Balliol College, University of Oxford, Oxford OX1 3BJ, United Kingdom; ¶ Department of Chemistry, Physical and Theoretical Chemistry Laboratory, University of Oxford, Oxford OX1 3QZ, United Kingdom

## Abstract

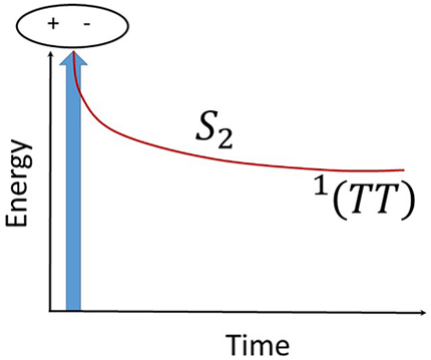

Internal conversion from the photoexcited state to a correlated singlet triplet-pair state is believed to be the precursor of singlet fission in carotenoids. We present numerical simulations of this process using a π-electron model that fully accounts for electron–electron interactions and electron–nuclear coupling. The time-evolution of the electrons is determined rigorously using the time-dependent density matrix renormalization group method, while the nuclei are evolved *via* the Ehrenfest equations of motion. We apply this to zeaxanthin, a carotenoid chain with 18 fully conjugated carbon atoms. We show that the internal conversion of the primary photoexcited state, *S*_2_, to the singlet triplet-pair state occurs adiabatically *via* an avoided crossing within ∼50 fs with a yield of ∼60%. We further discuss whether this singlet triplet-pair state will undergo exothermic versus endothermic intra- or interchain singlet fission.

The exotic electronic states of polyenes have been of abiding interest for nearly 50 years.^1−6^ Their fascinating properties arise because electron–electron (e–e) interactions and electron–nuclear (e–n) coupling are significantly enhanced in quasi-one-dimensional systems. One of the consequences of these interactions is that the lowest-energy excited singlet state is the nonemissive 2^1^*A*_*g*_^–^ state (labeled *S*_1_) that has significant correlated triplet-pair (or bimagnon) character. In contrast, the optically excited 1^1^*B*_*u*_^+^ state (labeled *S*_2_) has correlated electron–hole (or excitonic) character, and which—in the absence of e–e interactions and e–n coupling—would lie energetically below the 2^1^*A*_*g*_^–^ state. The energetic reversal of the bright (*S*_2_) and dark (*S*_1_) states has various photophysical consequences. For example, it explains the nonemissive properties of linear polyenes, it is responsible for the photoprotection properties of carotentoids in light harvesting complexes, and it is thought to be the cause of singlet fission in polyene-type systems.^7−16^

Singlet fission is a process by which a photoexcited state dissociates into two nongeminate triplets. In carotenoids and polyenes, while uncertainty remains as to whether the final step is an intra- or intermolecular process, the first step is understood to be the internal conversion of the photoexcited singlet, *S*_2_, into a correlated singlet triplet-pair state. In understanding the process of singlet fission, it is useful to recall how a pair of triplets combine,^15,16^ namely *T*_1_⊗*T*_1_ = *S* + *T* + *Q*, where *T*_1_ represents the lowest-energy triplet and *S*, *T*, and *Q* are the singlet, triplet, and quintet “correlated triplet-pair” states, respectively. Using the density matrix renormalization group (DMRG) method to solve the Pariser–Parr–Pople–Peierls (PPPP) model of π-conjugated systems, Valentine *et al.*(17) performed an extensive theoretical and computational study of the triplet-pair states of polyene chains. They showed, *via* the spin–spin correlation, bond dimerization, and triplet-pair overlaps, that the singlet triplet-pair state forms a band of states, 2^1^*A*_*g*_^–^, 1^1^*B*_*g*_^–^, 3^1^*A*_*g*_^–^, ..., each with different center-of-mass kinetic energies. In the long-chain limit, however, the kinetic energy of these low-energy states vanishes and their vertical energies converge to the same value. Importantly, this energy is ∼0.3 eV below the vertical energy of the quintet triplet-pair state. Because it was also shown^17^ that this quintet is an unbound pair of spin-correlated triplets, we can conclude that the triplet-pair binding energy in the singlet triplet-pair is ∼0.3 eV. (A similar conclusion concerning the binding energies of correlated triplet-pairs was made by Taffet *et al.*(18)) In addition, the vertical and relaxed energies of these low-energy singlet triplet-pair states lie below the vertical and relaxed energies of *S*_2_.

This picture becomes more complicated and interesting when we consider carotenoid chain lengths (i.e., *N* = 14–26, where *N* is the number of conjugated carbon atoms or twice the number of double bonds), as now the center-of-mass kinetic energy plays a role in the relative energetic ordering. In particular, it was shown in ref (17) that for all chain lengths the vertical and relaxed 2^1^*A*_*g*_^–^ energies lie below the corresponding 1^1^*B*_*u*_^+^ energies. The diabatic vertical and relaxed energies are illustrated in Figure 1 for the UV-Peierls model, defined in eq 4. In contrast, while the 1^1^*B*_*u*_^–^ relaxed energy is lower than the 1^1^*B*_*u*_^+^ relaxed energy for chain lengths *N* > 10, its vertical energy is higher than the 1^1^*B*_*u*_^+^ vertical energy for *N* ≤ 22 C atoms. Similarly, the relaxed 3^1^*A*_*g*_^–^ energy lies lower than the relaxed 1^1^*B*_*u*_^+^ energy for *N* ≥ 26, while its vertical energy is higher for *N* ≤ 42. These energetic orderings therefore imply that for certain chain lengths, because of diabatic energy level crossings, a vertical excitation to the 1^1^*B*_*u*_^+^ state will be followed by ultrafast internal conversion to either the 1^1^*B*_*u*_^–^ or 3^1^*A*_*g*_^–^ states.

**Figure 1:**
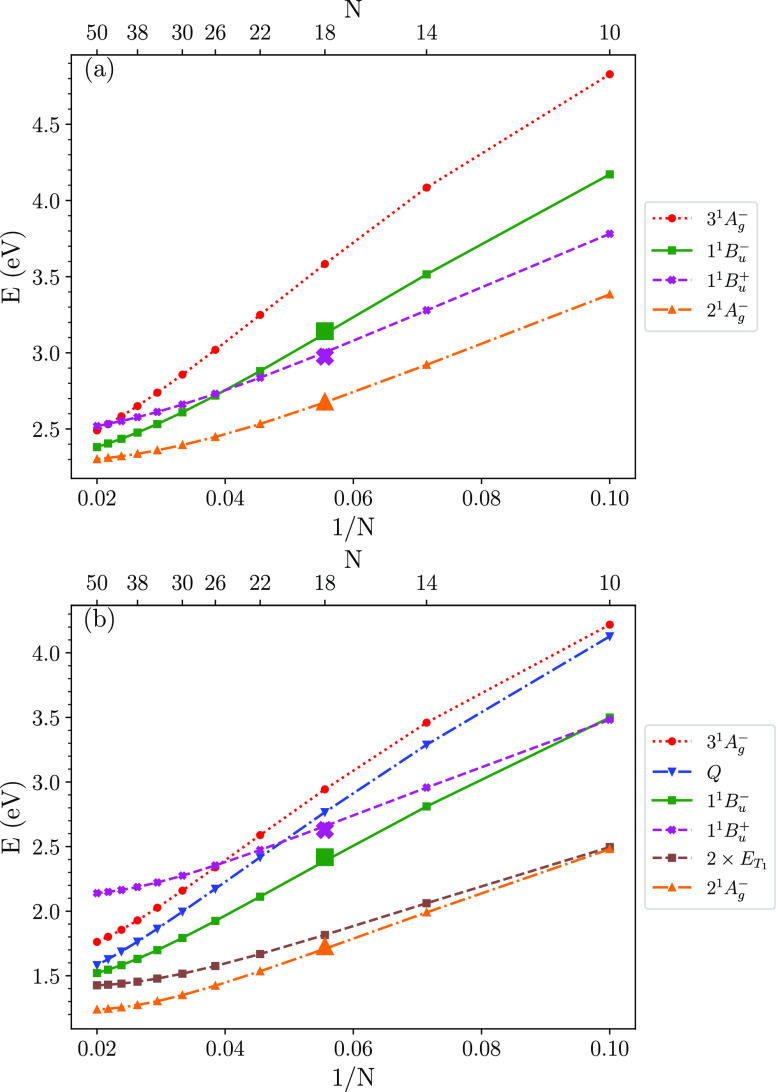
Vertical (a) and relaxed (b) diabatic singlet excitation energies of the UV-Peierls model (see eq 4). *N* is the number of conjugated carbon atoms, and *N*/2 is the number of double bonds. These results indicate that rapid internal conversion from 1^1^*B*_*u*_^+^ to 1^1^*B*_*u*_^–^ is energetically possible for 10 ≤ *N* ≤ 22, while rapid internal conversion from 1^1^*B*_*u*_^+^ to 3^1^*A*_*g*_^–^ is energetically possible for 26 ≤ *N* ≤ 42. Also shown in panel b is the quintet energy and twice the lowest triplet energy, implying that (i) singlet fission from 2^1^*A*_*g*_^–^ is endothermic for both intra- and intermolecular processes, (ii) singlet fission from 1^1^*B*_*u*_^–^ is endothermic for intramolecular and exothermic for intermolecular processes, and (iii) singlet fission from 3^1^*A*_*g*_^–^ is exothermic for both intra- and intermolecular processes. The large symbols shown at *N* = 18 are for the twisted zeaxanthin structure, indicating that its effective conjugation length is 18 C atoms (9 double bonds).

In addition to these diabatic energy level crossings, we observe that for the same chain length the relaxed 1^1^*B*_*u*_^–^ energy is lower than the relaxed quintet energy and *vice versa* for the 3^1^*A*_*g*_^–^ state. Finally, the relaxed energies of both the 1^1^*B*_*u*_^–^ and 3^1^*A*_*g*_^–^ states are more than twice the energy of the relaxed triplet (see Figure 1b). Thus, internal conversion to the 1^1^*B*_*u*_^–^ states implies potentially endothermic intramolecular singlet fission or exothermic intermolecular singlet fission. Conversely, internal conversion to the 3^1^*A*_*g*_^–^ state implies potentially exothermic intra- or intermolecular singlet fission.

These theoretical results (obtained using the Chandross–Mazumdar^19^ parametrization of the PPP model) are qualitatively consistent with the experimental observations on carotenoids summarized in Figure 1 of ref (20), with the difference being that experimentally the crossover in 3^1^*A*_*g*_^–^ and 1^1^*B*_*u*_^+^ relaxed energies occurs at 20 C atoms (i.e., 10 double bonds) rather than at 26 C atoms. The reader is referred to the excellent reviews^9,14^ of the electronic states of carotenoids.

In this work we investigate the internal conversion from the primary photoexcited singlet, *S*_2_, to the correlated singlet triplet-pair states in carotenoids. We perform rigorous dynamical simulations using a realistic model of π-electron conjugated systems that incorporates the key features of electron–electron repulsion and electron–nuclear coupling. The quantum system describing the electronic degrees of freedom is evolved *via* the time-dependent Schrödinger equation using the time-dependent DMRG (TD-DMRG) method. TD-DMRG is a very accurate method for simulating dynamics in highly correlated one-dimensional quantum systems.^21,22^ The nuclear degrees of freedom are treated classically *via* the Ehrenfest equations of motion. The decision to model the electronic dynamics *via* a π-electron model, rather than an *ab initio* electronic Hamiltonian, is a computational expediency motivated by the necessity of simulating a large, highly correlated electron system for long times (over 50 fs). The computational methods are described in section 2 of the Supporting Information. We refer the reader to static, *ab initio* DMRG-SCF calculations in polyenes^23^ and *ab initio* DMRG with perturbative corrections in carotenoids.^24,25^

As TD-DMRG is conveniently implemented with only on-site and nearest-neighbor Coulomb interactions, in this investigation the π-electron system is described by the extended Hubbard (or UV) model, defined by
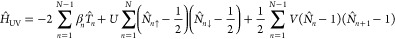
1Here, *T̂*_*n*_ = (1/2)∑_σ_ (*c*_*n*, σ_^†^*c*_*n*+1,σ_ + *c*_*n*+ 1,σ_^†^*c*_*n*, σ_) is the bond order operator and *N̂*_*n*_ is the number operator. *N* is the number of conjugated carbon-atoms (*N*/2 is the number of double bonds), β_*n*_ the electron hopping integral between neighboring C atoms, *U* the Coulomb interaction of two electrons in the same orbital, and *V* the nearest-neighbor Coulomb repulsion. Because the UV model does not contain the long-range Coulomb terms of the PPP model, as described in section 1 of the Supporting Information it is necessary to parametrize *U* and *V* to reproduce the predictions of ref (17).

The electrons couple to the nuclei *via* changes in the C–C bond length (which changes the effective electron transfer integral) *via*(6)
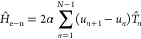
2where α is the electron–nuclear coupling parameter and *u*_*n*_ is the displacement of nucleus *n* from its undistorted position. (In principle, changes in the C–C bond length also change the nearest-neighbor Coulomb repulsion, *V*. However, as shown in ref (26), this effect is negligible.) Finally, the nuclear potential energy is described by
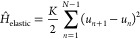
3where *K* is the nuclear spring constant.

The UV-Peierls Hamiltonian is now defined as
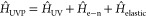
4This Hamiltonian is invariant under both a two-fold proper rotation (*i.e.*, a *C*_2*h*_ operation) and a particle-hole transformation (*i.e.*, (*N̂* – 1) → −(*N̂* – 1)), and so its eigenstates are labeled either *A*_*g*_^±^ or *B*_*u*_^±^. Internal conversion from *S*_2_ (*i.e.*, the nominal 1^1^*B*_*u*_^+^ state) to the triplet-pair singlets (with nominal negative particle-hole symmetry) is achieved *via* an interaction that breaks particle-hole symmetry. (Herein, we adopt the particle-hole notation used in ref (17), which is typically used by the experimental community. It is the opposite definition to that used in refs (6 and 26).) Carotenoids naturally possess such an interaction because of their methyl substituents, which act as electron donors to the π-system. (This is described in section 1.2 of the Supporting Information.) This symmetry-breaking term is
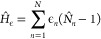
5which is odd under a particle-hole transformation.

In this work we investigate internal conversion in zeaxanthin, a carotenoid chain with 18 fully conjugated C atoms (and 9 double bonds) that plays a key role in biological photophysical processes^9,14^ and is thought to exhibit singlet fission.^27^ As shown in Figure 2, zeaxanthin possess *C*_2*h*_ symmetry, and thus, *Ĥ*_ϵ_ is even under this operation. [More correctly, because of its twisted end groups, zeaxanthin possesses *C*_2_ symmetry, and thus, the symmetry labels are *A* and *B*. However, in keeping with the common notation for carotenoids, we use the labels *A*_*g*_ and *B*_*u*_. In addition, because of the twisted end groups, the effective conjugation length is 9 double bonds (see Figure 1).^24^ As described in section 1.1 of the Supporting Information, we account for this by using a smaller value of β for the 2nd and 20th C–C bonds.] From both energetic and symmetry considerations, therefore, only 1^1^*B*_*u*_^+^ to 1^1^*B*_*u*_^–^ internal conversion is possible for this molecule.

**Figure 2:**
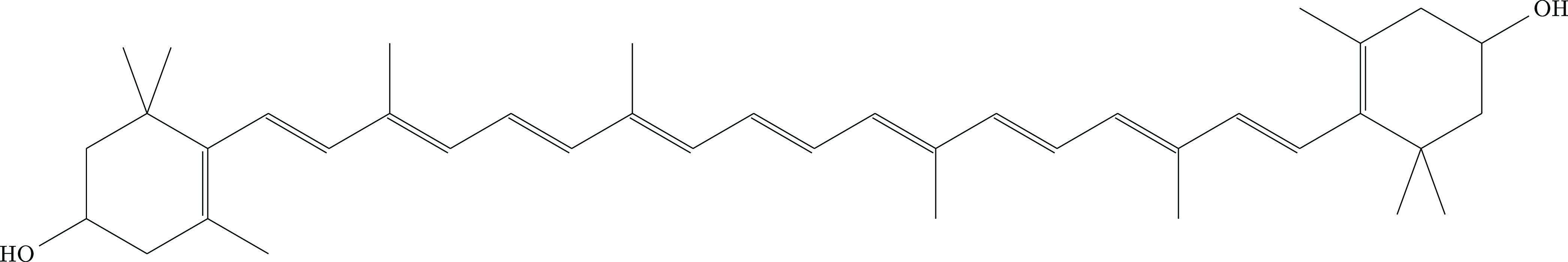
Structural formula of zeaxanthin. The end groups are twisted by 75° out of the plane of the molecule, thus reducing its effective conjugation length to 18 C atoms or 9 double bonds (see Figure 1).

We now define the *diabatic* states as eigenstates of *Ĥ*_UVP_, which thus have two-fold rotation and particle-hole symmetries. For our purposes the key diabatic states are 1^1^*B*_*u*_^–^ and 1^1^*B*_*u*_^+^. We define the *adiabatic* states as eigenstates of the full Born–Oppenheimer Hamiltonian, *Ĥ* = (*Ĥ*_UVP_ + *Ĥ*_ϵ_), and thus, these states are linear combinations of 1^1^*B*_*u*_^+^ and 1^1^*B*_*u*_^–^. As explained shortly, these states are *S*_2_ and *S*_3_.

For the purposes of our simulation, the initial state of the system at time *t* = 0, Ψ(*t* = 0), is taken to be the vertical excitation from the ground state to the dipole-allowed, second excited adiabatic singlet, *S*_2_. The ground state is obtained *via* static-DMRG^28^ solutions of the Hamiltonian *Ĥ* = (*Ĥ*_UVP_ + *Ĥ*_ϵ_) coupled to a Hellmann–Feynman iterator to determine the equilibrium ground state geometry.^26^ The system is subsequently described by the time-dependent wave function
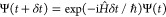
6evaluated using TD-DMRG (as described in section 2.2 of the Supporting Information).

We now describe the results of our simulations for zeaxanthin. At the Franck–Condon point the forces exerted on the nuclei from the electrons in the excited state, *S*_2_, causes *Ĥ*_e–n_ to change, which in turn causes an evolution of the electronic and nuclear degrees of freedom. (See section 2.3 of the Supporting Information for further details.) As the system evolves there is a crossover of the energies of the diabatic 1^1^*B*_*u*_^–^ and 1^1^*B*_*u*_^+^ states at ∼3 fs, as shown in Figure 3. The corresponding adiabatic energies (namely, the eigenvalues of the second and third excited singlet adiabatic states, *S*_2_ and *S*_3_), however, exhibit an avoided crossing, because the coupling between the diabatic states, ⟨1^1^*B*_*u*_^+^|*Ĥ*_ϵ_|1^1^*B*_*u*_^–^⟩, remains nonzero throughout the evolution. (The singlet ground and first excited adiabatic states, *S*_0_ and *S*_1_, are 1^1^*A*_*g*_ and 2^1^*A*_*g*_, respectively.) The avoided crossing is discussed in more detail in section 3 of the Supporting Information.

**Figure 3:**
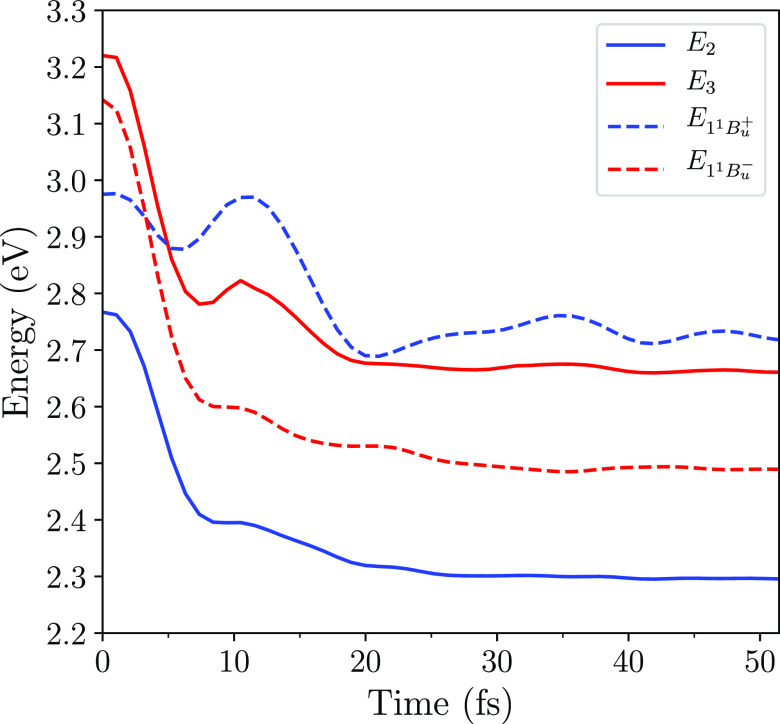
Excitation energies as a function of time of the diabatic 1^1^*B*_*u*_^–^ and 1^1^*B*_*u*_^+^ states (*i.e.*, eigenstates of *Ĥ*_UVP_), and the second and third excited adiabatic singlet states *S*_2_ and *S*_3_ (*i.e.*, eigenstates of (*Ĥ*_UVP_ + *Ĥ*_ϵ_)). These results are for zeaxanthin, shown in Figure 2. The initial condition is Ψ(0) = *S*_2_, the primary photoexcited state. These energies are found using the geometry determined by Ψ(*t*), whose evolution is determined by eq 6.

The evolution of the system described by Ψ(*t*) is illustrated in Figure 4, which shows the probabilities that it occupies *S*_2_ and *S*_3_. The initial condition is that Ψ(*t*) entirely occupies the lower adiabatic state *S*_2_, but around the avoided crossing at ∼5 fs this probability drops to ∼88% while the probability of occupying *S*_3_ rises to ∼12%. After ∼30 fs the probability that the system occupies *S*_2_ increases to over ∼95% and then remains essentially constant, indicating that this is an adiabatic transition. Similarly, the probability that the system occupies *S*_3_ reduces to less than 5%. As a consequence, the Ehrenfest approximation, which makes the erroneous assumption that the nuclei experience a mean force equal to the average from both adiabatic states,^29,30^ can be assumed to be largely valid here as only one state predominately determines the forces on the nuclei.

**Figure 4:**
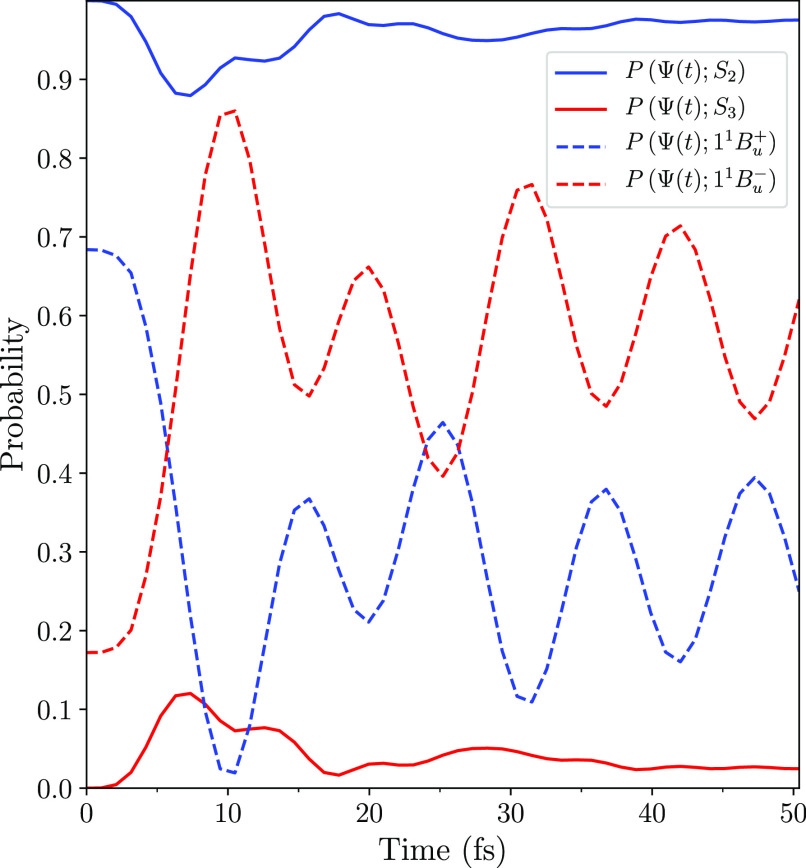
Probabilities that the system described by Ψ(*t*) occupies the excited adiabatic states *S*_2_ and *S*_3_, and the diabatic states 1^1^*B*_*u*_^+^ and 1^1^*B*_*u*_^–^. Note that Ψ(*t*) predominately evolves adiabatically on the surface of *S*_2_. The oscillations in the occupations of 1^1^*B*_*u*_^+^ and 1^1^*B*_*u*_^–^, with a period of 11 fs, are the nonstationary state oscillations described in the main text after eq 10.

Figure 5 shows the probabilities that the adiabatic states occupy the diabatic states, 1^1^*B*_*u*_^+^ and 1^1^*B*_*u*_^–^. Reflecting the crossover in the diabatic energies, at *t* = 0 the lower adiabatic state, *S*_2_, predominately occupies 1^1^*B*_*u*_^+^, while the upper adiabatic state, *S*_3_, predominately occupies 1^1^*B*_*u*_^–^. At the avoided crossing the adiabatic states are equal admixtures of both diabatic states. These probabilities then oscillate, before becoming damped after ∼40 fs. At this time *S*_2_ predominately occupies 1^1^*B*_*u*_^–^. As already noted, extensive calculations on polyenes^17^ indicate that the 1^1^*B*_*u*_^–^ state is the second member of the “2A_g_” family of correlated singlet triplet-pair states. In section 4 of the Supporting Information we confirm the triplet-pair character of these states in zeaxanthin *via* their bond dimerizations.

**Figure 5:**
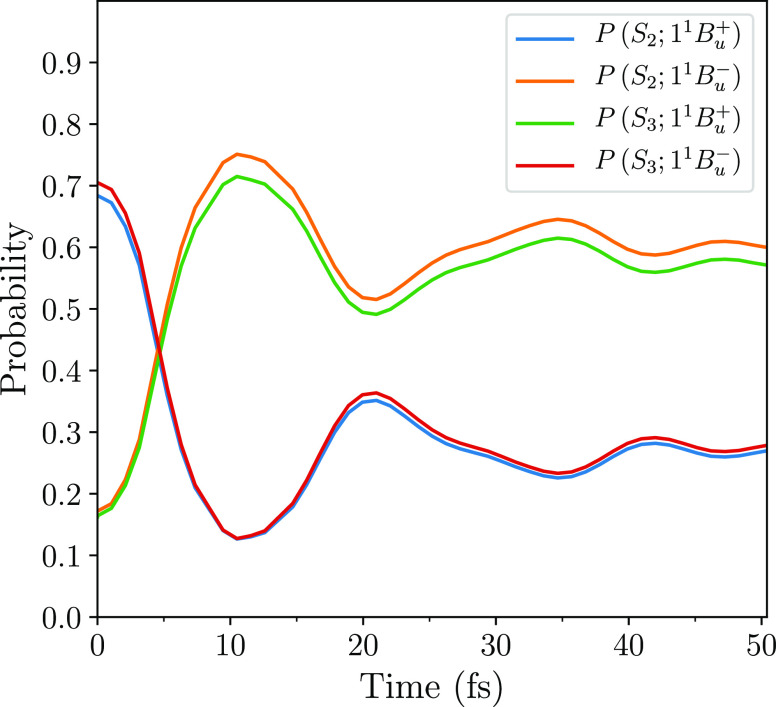
Probabilities that the adiabatic states, *S*_2_ and *S*_3_, occupy the diabatic states, 1^1^*B*_*u*_^+^ and 1^1^*B*_*u*_^–^. At time *t* = 0, *S*_2_ is the primary photoexcited state, which predominately occupies the exciton state, 1^1^*B*_*u*_^+^. Within 50 fs, *S*_2_ predominately occupies the triplet-pair state, 1^1^*B*_*u*_^–^, although it retains some exciton component. The oscillations in the probabilities with a period of ∼20 fs coincide with the period of the C–C bond vibration.

As shown in Figure 4, Ψ(*t*) is entirely composed of the adiabatic states *S*_2_ and *S*_3_. In addition, the adiabatic probabilities and energies become quasi-stationary after ∼30 fs. Thus, we can adopt a two-level system and express Ψ(*t*) as the nonstationary state
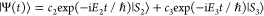
7where the probability amplitudes, *c*_2_ and *c*_3_, are assumed to be constant. Similarly, Figure 5 shows that the adiabatic states are ∼90% composed of the diabatic states 1^1^*B*_*u*_^+^ and 1^1^*B*_*u*_^–^, *i.e.*,
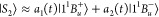
8and
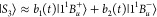
9where |*a*_1_(*t*)|^2^ ≈ |*b*_2_(*t*)|^2^ and |*a*_2_(*t*)|^2^ ≈ |*b*_1_(*t*)|^2^. Thus, the probability that the system occupies the singlet triplet-pair state, *P*(Ψ(*t*), 1^1^*B*_*u*_^–^) = |⟨Ψ(*t*)|1^1^*B*_*u*_^–^⟩|^2^, is
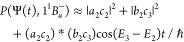
10This probability is illustrated in Figure 4 by the dashed-red curve. For *t* ≳ 30 fs it oscillates with a period *T* = *h*/(*E*_3_ – *E*_2_) = 11 fs, showing that eqs 7 and 10 are valid.

In general, as well as causing oscillations in *P*(Ψ(*t*),1^1^*B*_*u*_^–^), the quantum coherences between the adiabatic states cause time-dependent observables. In practice, however, interactions of the carotenoid chain (*i.e.*, the system) with its surroundings will cause decoherence, and in particular the oscillations in the probability that the system occupies the diabatic states 1^1^*B*_*u*_^–^ and 1^1^*B*_*u*_^+^ will be damped. These processes are not completely modeled by our Ehrenfest approximation of the nuclear degrees of freedom, so we estimate the singlet triplet-pair yield by the “classical” component of eq 10, *i.e.*, *P*_classical_ = |*a*_2_ *c*_2_|^2^ + |*b*_2_ *c*_3_|^2^. This yield is ∼60% after ∼50 fs.

We now summarize the results of our simulations. At the Franck–Condon point at *t* = 0, the system is prepared in the primary photoexcited state, *i.e.*, the second adiabatic state, *S*_2_. At this time *S*_2_ is predominately the exciton state, 1^1^*B*_*u*_^+^. The system then predominately evolves adiabatically on the potential energy surface of *S*_2_, avoiding an energy level crossing with *S*_3_ at ∼5 fs, such that within 50 fs *S*_2_ is now predominately composed of the triplet-pair state, 1^1^*B*_*u*_^–^. We note, however, that there is also a ∼25% probability that *S*_2_ occupies 1^1^*B*_*u*_^+^ and therefore *S*_2_ does not evolve to a completely dark state. The third adiabatic state, *S*_3_, is the complement of *S*_2_, namely at *t* = 0 it is predominately 1^1^*B*_*u*_^–^, while at 50 fs it is predominately 1^1^*B*_*u*_^+^, with a small component 1^1^*B*_*u*_^–^. According to our earlier work (see Figure 9 of ref (17)), the ultrafast internal conversion from *S*_2_ to 1^1^*B*_*u*_^–^ (or more generally, to the “2A_g_” family of singlet triplet-pair states) implies an ultrafast generation of a strong excited-state absorption of ∼2.4 eV (this transition energy is the same as the *T*_1_ → *T*_*n*_ transition energy^17^), which is consistent with experimental observations.^9^

As we have already noted, the relaxed 1^1^*B*_*u*_^–^ state lies lower in energy than the relaxed quintet triplet-pair state (by ∼0.4 eV in zeaxanthin), and as this quintet corresponds to a pair of spin-correlated but unbound triplets,^17^ we can conclude that potential intramolecular singlet fission *via* 1^1^*B*_*u*_^–^ is endothermic. As Figure 1b indicates, however, intermolecular singlet fission *via* 1^1^*B*_*u*_^–^ on two carotenoid molecules of the same length is an exothermic process (by ∼0.6 eV in zeaxanthin), because of an increase in (negative) nuclear reorganization energy and a decrease in (positive) confinement energy for single triplets on a chain.

Potential intramolecular singlet fission *via* 3^1^*A*_*g*_^–^ is exothermic, because its excess kinetic energy overcomes the triplet binding energy. Indeed, as the polyene chain length increases, internal conversion from 1^1^*B*_*u*_^+^ occurs to higher kinetic energy members of the “2A_g_” family, meaning that for *N* > 26 all internal conversion is energetically favorable for intramolecular singlet fission. In practice, because 1^1^*B*_*u*_^–^ and 3^1^*A*_*g*_^–^ are higher quasi-momentum counterparts of 2^1^*A*_*g*_^–^, phonon-mediated internal conversion from the former to the latter is possible. Alternatively, a vibronically allowed internal conversion from *S*_2_ to 2^1^*A*_*g*_^–^ might occur. We note, however, that singlet fission from 2^1^*A*_*g*_^–^ is expected to be endothermic for both intra- and intermolecular processes. (This is a robust prediction over a wide range of model parameters, as indicated by Figure 7.4 of ref (6).)

In conclusion, we have performed dynamical simulations of the primary photoexcited state, *S*_2_, of cartotenoids using a π-electron model that fully accounts for electron–electron interactions and electron–nuclear coupling. The time-evolution of the electrons was determined rigorously using the time-dependent density matrix renormalization method, while the nuclei were evolved *via* the Ehrenfest equations of motion. For zeaxanthin, we showed that internal conversion to a singlet triplet-pair state (*i.e.*, 1^1^*B*_*u*_^–^) occurs adiabatically *via* an avoided crossing within 50 fs with a yield of ∼60%. However, *S*_2_ still retains some excitonic character (*i.e.*, 1^1^*B*_*u*_^+^). We further predict that only intermolecular exothermic singlet fission is possible for shorter carotenoids, but intramolecular exothermic singlet fission is possible for longer chains.

Although our theoretical predictions—determined using the Chandross-Mazumdar^19^ parametrization of the PPP model—are consistent with a wide range of experimental observations,^9,14,20,31^ we note that there does not exist a settled consensus about the relative orderings of the vertical energies of the singlet triplet-pair states and *S*_2_, with some authors^24,25^ arguing that the vertical energy of the 2^1^*A*_*g*_^–^ state is higher than that of the 1^1^*B*_*u*_^+^ state. Because the 1^1^*B*_*u*_^+^ state is excitonic and thus is a fluctuating electric dipole,^32^ its energy is strongly affected by the polarizability of the carotenoid’s core electrons and its environment. This implies that in some environments the vertical 2^1^*A*_*g*_^–^ energy might lie higher than the vertical 1^1^*B*_*u*_^+^ energy and *vice versa* for their relaxed energies, and therefore, rapid internal conversion is possible from the 1^1^*B*_*u*_^+^ state directly to the 2^1^*A*_*g*_^–^ state.

Future work will investigate internal conversion from *S*_2_ to singlet triplet-pair states (including the 2^1^*A*_*g*_^–^ state) for carotenoids of different lengths and with no definite spatial symmetry. To make a better connection with experimental observables, we will also compute the transient absorption. Finally, we will investigate the role of bond rotations and examine the validity of the Ehrenfest approximation by quantizing the phonon degrees of freedom.

## Supporting Information Available

The following technical sections: (1) parametrization of the Hamiltonian, (2) computational methods, (3) dynamics at the avoided crossing, and (4) bond dimerizations (PDF)
Transparent Peer Review report available (PDF)
