# Supplementary material for: Singlet Triplet-Pair Production and Possible Singlet-Fission in Carotenoids
Source: J Phys Chem Lett. 2022 Feb 2;13(5):1344–9. doi: 10.1021/acs.jpclett.1c03812 (PMC9084603; doi:10.1021/acs.jpclett.1c03812)
Supplement: Supplementary file 1 — jz1c03812_si_001.pdf [file jz1c03812_si_001.pdf]

Singlet Triplet-Pair Production and Possible Singlet-Fission in Carotenoids Supporting Information

Dilhan Manawadu,[*,†] Darren Valentine,[‡] Max Marcus,[¶] and William Barford[*,¶]

†*Department of Chemistry, Physical and Theoretical Chemistry Laboratory, University of Oxford, Oxford, OX1 3QZ, United Kingdom*

Linacre College, University of Oxford, Oxford, OX1 3JA, United Kingdom

‡*Department of Chemistry, Physical and Theoretical Chemistry Laboratory, University of Oxford, Oxford, OX1 3QZ, United Kingdom*

Balliol College, University of Oxford, Oxford, OX1 3BJ, United Kingdom

¶*Department of Chemistry, Physical and Theoretical Chemistry Laboratory, University of Oxford, Oxford, OX1 3QZ, United Kingdom*

E-mail: dilhan.manawadu@chem.ox.ac.uk; william.barford@chem.ox.ac.uk

Table of contents:

1. Parametrization of the Hamiltonian

2. Computational methods

3. Dynamics at the avoided crossing

4. Bond dimerization

1. Parametrization of the Hamiltonian

1.1 Parametrization of $H_{\mathbf{UV}}$

As described in Section 2.2, efficient implementation of the time-dependent density matrix renormalization group method via the Trotter decomposition of the evolution operator requires that the Hamiltonian be partitioned into a sum of bond Hamiltonians. This implies that only on-site and nearest neighbor terms can be retained in the Hamiltonian. Thus, the purely electronic component of the Hamiltonian is the extended-Hubbard (or UV) model. This model, defined by

$$\hat{H}_{\text{UV}} = -2\sum_{n=1}^{N-1}\beta_n\hat{T}_n + U\sum_{n=1}^{N}\left(\hat{N}_{n\uparrow} - \frac{1}{2}\right)\left(\hat{N}_{n\downarrow} - \frac{1}{2}\right) + \frac{1}{2}\sum_{n=1}^{N-1}V\left(\hat{N}_n - 1\right)\left(\hat{N}_{n+1} - 1\right), \quad (1)$$

contains a nearest neighbor electron transfer term, β_n, and onsite and nearest neighbor Coulomb interactions, U and V, respectively. $\hat{T}_n = \frac{1}{2}\sum_\sigma\left(c^\dagger_{n,\sigma}c_{n+1,\sigma} + c^\dagger_{n+1,\sigma}c_{n,\sigma}\right)$ is the bond order operator, $\hat{N}_n$ is the number operator and N (= 22 for zeaxanthin) is the number of conjugated carbon-atoms ($N/2$ is the number of double bonds).

In recent work[1] we studied the excited states of conjugated polyenes using the Pariser-Parr-Pople-Peierls (PPPP) model. The purely electronic component of this Hamiltonian, namely, the Pariser-Parr-Pople (PPP) model, contains nearest neighbor electron transfer terms and long range Coulomb interactions. We used the condensed-phase Chandross-Mazumdar[2] parametrization of the PPP model.

In the current investigation we parametrized the UV model so that it replicates the predictions of the PPPP model with the Chandross-Mazumdar[2] parametrization. We found that keeping $\beta = 2.4$ eV, $\omega_0 = \sqrt{K/m} = 2.15 \times 10^{14}$ s^{-1} and $K = 46$ eV $\AA^{-2}$ as before, and choosing $U = 7.25$ eV, $V = 2.75$ eV and $\alpha = 4.6$ eV $\AA^{-1}$ gives quantitatively equivalent results, as shown by comparing Fig. 1 of the main paper with Figures 2 and 3 of Ref.[1]

We next modeled the twist of the end groups in zeaxanthin by adjusting the electron

transfer integrals for 2nd and 20th bonds (i.e., $C_2{-}C_3$ and $C_{20}{-}C_{21}$, as shown in Fig. 1) according to $\beta_n = \beta \cos \phi_n$, where $\phi_n = 75^0$ is the dihedral angle for these bonds.[3]

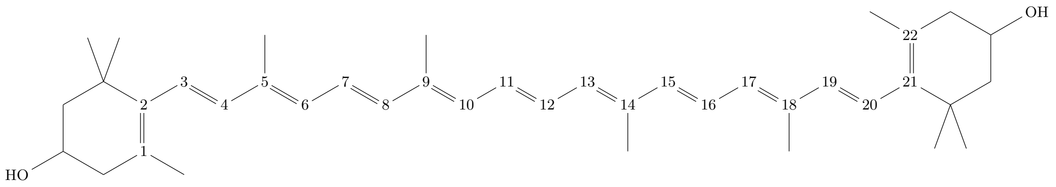

Figure 1: Structural formula of zeaxanthin. The end groups are twisted by 75^0 out of the plane of the molecule.

The diabatic energies for zeaxanthin are indicated by the large symbols in Fig. 1 of the main paper.

1.2 Parametrization of $\hat{H}_\epsilon$

The substituents on carotenoid molecules causes the particle-hole symmetry of polyene chains to be broken, as they act as electron donors to the π-system which implies that the π-electron Mulliken charge densities[4] are not equal to unity on every carbon-atom. The Mulliken charge densities for zeaxanthin were calculated using the ORCA program package.[5,6] To that end a geometry optimisation was performed using density functional theory (DFT) with a B3LYP functional[7] and a def2-TZVP basis set[8,9] before calculating the electron densities. The tabulated values, shown in Table I, are the π-electron charges associated with each carbon atom along the conjugated backbone, numbered 1-22 in Fig. 1.

We ensure that our π-electron model replicates the DFT-calculated charges (subject to charge neutrality) by supplementing $\hat{H}_{\mathrm{UV}}$ with

$$\hat{H}_\epsilon = \sum_{n=1}^{N} \epsilon_n (\hat{N}_n - 1), \tag{2}$$

where the potential energy terms are parametrized to replicate the DFT calculations. These are listed in Table I.

Table 1: The π-electron Mulliken charges from the *ab initio* DFT calculation and the parameters for $\hat{H}_\epsilon$. In order to maintain π-electron charge neutrality, each *ab initio* charge was reduced by $0.07q$. $\epsilon_n = +\epsilon_{N+1-n}$ for even $\hat{H}_\epsilon$, implying a $1^1B_u^+$ to $1^1B_u^-$ crossover. The carbon-sites are labelled in Fig. 1.

C-site, n	Mulliken charges (q)	ϵ_n (eV)
1	1.34	-3.6
2	0.94	-1.0
3	1.04	0.0
4	0.97	0.0
5	1.22	-1.6
6	0.98	-0.6
7	1.05	0.0
8	0.98	0.0
9	1.22	-1.6
10	0.98	-0.6
11	1.03	0.0

2. Computational Methods

The computational technique that underpins our calculations of the highly-correlated π-electronic system is the density matrix renomalization group (DMRG) method, introduced by White for static problems in 1992.[10] DMRG is most readily suited to reduced single-particle basis Hamiltonians, such as the π-electron PPP model, for which DMRG converges to a specified accuracy for a finite-size Hilbert space. The use of a reduced single-particle basis is not necessarily a disadvantage over *ab initio* methods, as when correctly parameterized the PPP model makes very accurate predictions.[2,11] As such, since the first use of DMRG in quantum chemistry in 1997,[12] DMRG has been widely used to investigate the electronic properties of conjugated polymers using the PPP model.[13–19] Recently, more *ab initio* Hamiltonians have also been used for polyenes[20] and carotenoids.[21,22] The extension of DMRG to time-dependent DMRG for dynamical problems was made in 2004.[23,24] (See Ref[25] for a recent review of applications of DMRG in quantum chemistry.)

2.1 Determining the ground and excited states

The dimerized ground state is determined by the full Born-Oppenheimer Hamiltonian, defined by

$$\hat{H} = \hat{H}_{\mathrm{UVP}} + \hat{H}_{\epsilon}, \tag{3}$$

where

$$\hat{H}_{\mathrm{UVP}} = \hat{H}_{\mathrm{UV}} + 2\alpha \sum_{n=1}^{N-1} (u_{n+1} - u_n)\,\hat{T}_n + \frac{K}{2} \sum_{n=1}^{N-1} (u_{n+1} - u_n)^2 \tag{4}$$

is the UV-Peierls Hamiltonian. Equation (3) is solved for fixed nuclear displacements, u_n, using the static DMRG method. The equilibrium displacements are found by iterative application of Eq. (10) with $f_n = 0$.[16]

The vertical and relaxed diabatic energies (i.e., $\hat{H}_{\epsilon} = 0$), shown in Fig. 1 of the main paper, are also obtained via the static-DMRG method.[16]

2.2 Solution of the time-dependent Schrödinger equation

The dynamics of the evolving system is fully determined by solving the time-dependent Schrödinger equation

$$\mathrm{i}\hbar \frac{\mathrm{d}|\Psi(t)\rangle}{\mathrm{d}t} = \hat{H}|\Psi(t)\rangle, \tag{5}$$

where $\hat{H}$ is the full Hamiltonian, defined in Eq. (3). Equation (5) has the formal solution

$$|\Psi(t + \delta t)\rangle = \exp(-\mathrm{i}\hat{H}\delta t/\hbar)|\Psi(t)\rangle, \tag{6}$$

where the initial condition is $|\Psi(t = 0)\rangle = |S_2\rangle$. $|S_2\rangle$, the primary photoexcited state, is the second adiabatic vertically excited singlet state from the dimerized groundstate.

$|\Psi(t)\rangle$ is determined using the adaptive time-dependent density matrix renormalisation group (TD-DMRG) method, where the truncated Hilbert space is updated such that loss of information about the system is minimized.[23,24] Since the full Hamiltonian only contains

on-site and nearest neighbor terms, it can be written as a sum of bond Hamiltonians, i.e.,

$$\hat{H} = \hat{H}_{1,2} + \hat{H}_{2,3} + \hat{H}_{3,4} + \cdots + \hat{H}_{n,n+1} + \cdots + \hat{H}_{N-1,N}, \tag{7}$$

where $\hat{H}_{n,n+1}$ acts on the nth. bond. A Suzuki-Trotter decomposition is invoked for the propagator, i.e.,

$$\mathrm{e}^{-\mathrm{i}\hat{H}\delta t/\hbar} \approx \mathrm{e}^{-\mathrm{i}\hat{H}_{1,2}\delta t/2\hbar}\mathrm{e}^{-\mathrm{i}\hat{H}_{2,3}\delta t/2\hbar}\cdots\mathrm{e}^{-\mathrm{i}\hat{H}_{N-1,N}\delta t/2\hbar}\mathrm{e}^{-\mathrm{i}\hat{H}_{N-1,N}\delta t/2\hbar}\cdots\mathrm{e}^{-\mathrm{i}\hat{H}_{2,3}\delta t/2\hbar}\mathrm{e}^{-\mathrm{i}\hat{H}_{1,2}\delta t/2\hbar} + O(\delta t^3) \tag{8}$$

The propagator is applied $t/\delta t$ times on $|\Psi(t=0)\rangle$ to find $|\Psi(t)\rangle$ with $\delta t = 3 \times 10^{-4}$ fs.

2.3 Ehrenfest equations of motion

The nuclear degrees of freedom are treated classically via the Ehrenfest equations of motion. The force on atom n is

$$f_n = -\frac{\mathrm{d}\langle\Psi|\hat{H}|\Psi\rangle}{\mathrm{d}u_n} = -\left\langle \frac{\mathrm{d}\hat{H}}{\mathrm{d}u_n} \right\rangle, \tag{9}$$

i.e.,

$$f_n = 2\alpha\left(\langle\hat{T}_n\rangle - \langle\hat{T}_{n-1}\rangle\right) - K\left(2u_n - u_{n+1} - u_{n-1}\right). \tag{10}$$

The nuclei obey the coupled equations of motion

$$\frac{\mathrm{d}u_n(t)}{\mathrm{d}t} = \frac{p_n(t)}{m} \tag{11}$$

and

$$\frac{\mathrm{d}p_n(t)}{\mathrm{d}t} = f_n(t) - \gamma p_n(t), \tag{12}$$

where a phenomenological linear damping term γp_n is introduced to cause relaxation of the nuclei. The equations of motion are propagated using the damped Velocity Verlet scheme

(derived in Appendix 5.A of Ref[26]), i.e.,

$$u_n(t+\Delta t) = u_n(t) + \frac{p_n(t)}{m}\Delta t + \frac{1}{2}\frac{(f_n(t) - \gamma p_n(t))}{m}\Delta t^2 \tag{13}$$

and

$$p_n(t+\Delta t) = \frac{1}{(1+\gamma\Delta t/2)}\left((1-\gamma\Delta t/2)p_n(t) + \frac{\Delta t}{2}\left(f_n(t+\Delta t) + f_n(t)\right)\right). \tag{14}$$

We use $\gamma = 1.52 \times 10^{14}$ s^{-1}, corresponding to $\gamma = \omega_D/2 = \omega/\sqrt{2}$.The integration time step is set to $\Delta t = 0.003$ fs such that $\Delta t' = \omega_D \Delta t = 9 \times 10^{-4} \ll 1$.

2.4 Accuracy and convergence tests

Due to the variational nature of the DMRG algorithm, its accuracy can be systematically improved by increasing the Hilbert space. Convergence of the closely related Pariser-Parr-Pople-Peierls model has been extensively studied.[15,17–19]

In general, in TD-DMRG the number of states required to represent a time-evolving state vector accurately is given by

$$m = 2^S, \tag{15}$$

where S is the von Neumann entanglement entropy. For our simulations, the maximum entropy reached by a block is $S_{\max} = 2.46$. We typically retain over 400 states per block, which is much more than the number of states required by Eq. (15). We also checked the accuracy of our TD-DMRG calculations by confirming that the vertically excited diabatic states dynamically evolve to the relaxed configurations determined by the static DMRG described in Section 2.1.

3. Dynamics at the Avoided Crossing

Fig. 4 of the main paper shows that $\Psi(t)$ is entirely composed of the adiabatic states S_2 and S_3 (the second and third excited singlet states of the full Hamiltonian, $\hat{H} = \hat{H}_{\text{UVP}} + \hat{H}_\epsilon$).

In addition, the adiabatic probabilities and energies become quasi-stationary after ~ 30 fs. Thus, we can adopt a two-level system and express $\Psi(t)$ as the non-stationary state

$$|\Psi(t)\rangle = c_2 \exp(-\mathrm{i}E_2 t/\hbar)|S_2\rangle + c_3 \exp(-\mathrm{i}E_3 t/\hbar)|S_3\rangle, \tag{16}$$

where the probability amplitudes, c_2 and c_3, are assumed to be constant. Similarly, Fig. 5 of the main paper shows that the adiabatic states are $\sim 90\%$ composed of the diabatic states $1^1B_u^+$ and $1^1B_u^-$ (eigenstates of the UV-Peierls Hamiltonian, $\hat{H}_{\mathrm{UVP}}$), i.e.,

$$|S_2\rangle \approx a_1(t)|1^1B_u^+\rangle + a_2(t)|1^1B_u^-\rangle \tag{17}$$

and

$$|S_3\rangle \approx b_1(t)|1^1B_u^+\rangle + b_2(t)|1^1B_u^-\rangle. \tag{18}$$

In a two-level system, $|a_1|^2 = |b_2|^2$ and $|a_2|^2 = |b_1|^2$. As shown by Fig. 5 of the main paper, however, these conditions are not exactly satisfied, so our system is not precisely a two-level system.

The evolution of $|\Psi(t)\rangle$ is determined by $(\hat{H}_{\mathrm{UVP}} + \hat{H}_\epsilon)$. $\hat{H}_{\mathrm{UVP}}$ is even under both a C_{2h} operation and a particle-hole transformation, whereas $\hat{H}_\epsilon$ is odd under a particle-hole transformation and (in our model) even under a C_{2h} operation. Thus, in the 2×2 basis of the diabatic states $1^1B_u^+$ and $1^1B_u^-$ the Hamiltonian is block-diagonalized by $\hat{H}_{\mathrm{UVP}}$ and $\hat{H}_\epsilon$.

Therefore, the Hamiltonian matrix is

$$\begin{pmatrix} E_{1^1B_u^+} & V \\ V^* & E_{1^1B_u^-} \end{pmatrix}, \tag{19}$$

where $E_X = \langle X|\hat{H}_{\mathrm{UVP}}|X\rangle$ and $V = \langle 1^1B_u^+|\hat{H}_\epsilon|1^1B_u^-\rangle$. The instantaneous eigenvalues are

$$E_\pm = \frac{E_{1^1B_u^+} + E_{1^1B_u^-}}{2} \pm \left(\left(\frac{E_{1^1B_u^+} - E_{1^1B_u^-}}{2} \right)^2 + |V|^2 \right)^{1/2}, \tag{20}$$

where we associate E_2 with E_- and E_3 with E_+.

The coupling matrix element, V, remains finite during the evolution and so at the energy crossing of the diabatics (i.e., when $E_{1^1B_u^+}{=}E_{1^1B_u^-}$) there is an avoided crossing with a gap $\Delta E = (E_+ - E_-) = 2|V| = 0.38$ eV, as can be seen in Fig. 3 of the main paper.

4. Bond Dimerization

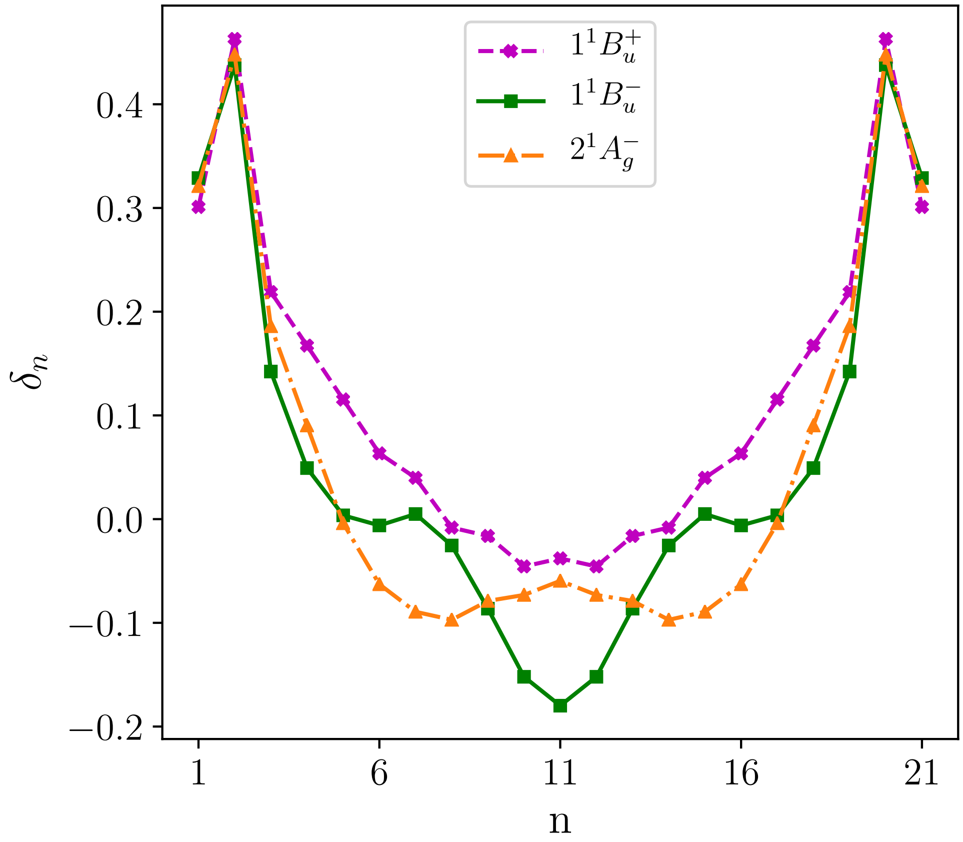

Figure 2: The staggered bond dimerization, δ_n, versus bond index, n, for zeaxanthin of the $2^1A_g^-$, $1^1B_u^-$ and $1^1B_u^+$ states, indicating the four-soliton structure of the $2^1A_g^-$ and $1^1B_u^-$ triplet-pair states.

Energetic considerations, as well as calculations of spin-spin correlation, bond dimerization and triplet-pair overlaps in polyenes[1] indicate that the $1^1B_u^-$ state is the second member of the '$2A_g$' family of correlated singlet triplet-pair states. Here, we confirm in Fig. 2 the triplet-pair character of these states in zeaxanthin via their staggered bond dimerizations,

defined by $\delta_n = (-1)^n(\delta u_n - \bar{\delta u})/\bar{\delta u}$ (where $\bar{\delta u}$ is the average value of δu_n). These match those of Fig. 4 of Ref.[1] for a polyene of 54 C-atoms (27 double bonds). In particular, the $2^1A_g^-$ and $1^1B_u^-$ states exhibit the four-soliton structure of triplet-pair states, and in addition the $2^1A_g^-$ exhibits the characteristic 'double potential' of a pair of bound triplets.

References

(1) Valentine, D. J.; Manawadu, D.; Barford, W. Higher-energy triplet-pair states in polyenes and their role in intramolecular singlet fission. *Physical Review B* **2020**, *102*, 125107.

(2) Chandross, M.; Mazumdar, S. Coulomb interactions and linear, nonlinear, and triplet absorption in poly(para-phenylenevinylene). *Physical Review B* **1997**, *55*, 1497.

(3) Bartalucci, G.; Coppin, J.; Fisher, S.; Hall, G.; Helliwell, J. R.; Helliwell, M.; Liaaen-Jensen, S. Unravelling the chemical basis of the bathochromic shift in the lobster carapace; new crystal structures of unbound astaxanthin, canthaxanthin and zeaxanthin. *Acta Crystallographica Section B-Structural Science Crystal Engineering and Materials* **2007**, *63*, 328.

(4) Mulliken, R. S. Electronic Population Analysis on LCAO–MO Molecular Wave Functions. I. *Journal of Chemical Physics* **1955**, *23*, 1833.

(5) Neese, F. The ORCA program system. *Wiley Interdisciplinary Reviews: Computational Molecular Science* **2012**, *2*, 73.

(6) Neese, F. Software update: the ORCA program system, version 4.0. *Wiley Interdisciplinary Reviews: Computational Molecular Science* **2017**, *8*, e1327.

(7) Stephens, P. J.; Devlin, F. J.; Chabalowski, C. F.; Frisch, M. J. Ab Initio Calculation of Vibrational Absorption and Circular Dichroism Spectra Using Density Functional Force Fields. *J. Phys. Chem.* **1994**, *98*, 11623.

(8) Weigend, F.; Ahlrichs, R. Balanced basis sets of split valence, triple zeta valence and quadruple zeta valence quality for H to Rn: Design and assessment of accuracy. *Phys. Chem. Chem. Phys.* **2005**, *7*, 3297.

(9) Weigend, F.; Ahlrichs, R. Accurate Coulomb-fitting basis sets for H to Rn. *Phys. Chem. Chem. Phys.* **2006**, *8*, 1057.

(10) White, S. R. Density-Matrix Formulation for Quantum Renormalization-Groups. *Physical Review Letters* **1992**, *69*, 2863.

(11) Castleton, C. W. M.; Barford, W. Screening and the quantitative π-model description of the optical spectra and polarizations of phenyl based oligomers. *Journal of Chemical Physics* **2002**, *117*, 3570.

(12) Barford, W.; Bursill, R. J. Theory of molecular excitons in the phenyl-based organic semiconductors. *Chemical Physics Letters* **1997**, *268*, 535.

(13) Yaron, D.; Moore, E. E.; Shuai, Z.; Bredas, J. L. Comparison of density matrix renormalization group calculations with electron-hole models of exciton binding in conjugated polymers. *Journal of Chemical Physics* **1998**, *108*, 7451.

(14) Fano, G.; Ortolani, F.; Ziosi, L. The density matrix renormalization group method: Application to the PPP model of a cyclic polyene chain. *Journal of Chemical Physics* **1998**, *108*, 9246.

(15) Bursill, R. J.; Barford, W. Electron-lattice relaxation, and soliton structures and their interactions in polyenes. *Physical Review Letters* **1999**, *82*, 1514.

(16) Barford, W.; Bursill, R. J.; Lavrentiev, M. Y. Density-matrix renormalization-group calculations of excited states of linear polyenes. *Physical Review B* **2001**, *63*, 195108.

(17) Barford, W.; Bursill, R. J.; Lavrentiev, M. Y. Breakdown of the adiabatic approximation in trans-polyacetylene. *Physical Review B - Condensed Matter and Materials Physics* **2002**, *65*.

(18) Bursill, R. J.; Barford, W. Large-scale numerical investigation of excited states in

poly(para-phenylene). *Physical Review B - Condensed Matter and Materials Physics* **2002**, *66*, 1.

(19) Bursill, R. J.; Barford, W. Symmetry-adapted density matrix renormalization group calculations of the primary excited states of poly(para-phenylene vinylene). *Journal of Chemical Physics* **2009**, *130*, 19548722.

(20) Hu, W. F.; Chan, G. K. L. Excited-State Geometry Optimization with the Density Matrix Renormalization Group, as Applied to Polyenes. *Journal of Chemical Theory and Computation* **2015**, *11*, 3000.

(21) Taffet, E. J.; Lee, B. G.; Toa, Z. S. D.; Pace, N.; Rumbles, G.; Southall, J.; Cogdell, R. J.; Scholes, G. D. Carotenoid Nuclear Reorganization and Interplay of Bright and Dark Excited States. *Journal of Physical Chemistry B* **2019**, *123*, 8628.

(22) Khokhlov, D.; Belov, A. Ab Initio Study of Low-Lying Excited States of Carotenoid-Derived Polyenes. *Journal of Physical Chemistry A* **2020**, *124*, 5790.

(23) White, S. R.; Feiguin, A. E. Real-time evolution using the density matrix renormalization group. *Physical Review Letters* **2004**, *93*, 076401.

(24) Daley, A. J.; Kollath, C.; Schollwöck, U.; Vidal, G. Time-dependent density-matrix renormalization-group using adaptive effective Hilbert spaces. *Journal of Statistical Mechanics: Theory and Experiment* **2004**, *2004*, P04005.

(25) Baiardi, A.; Reiher, M. The density matrix renormalization group in chemistry and molecular physics: Recent developments and new challenges. *Journal of Chemical Physics* **2020**, *152*, 040903.

(26) Valentine, D. *Singlet fission in linear π-conjugated systems*; DPhil thesis, University of Oxford: Oxford, 2020.
