# Supplementary material for: Singlet Triplet-Pair Production and Possible Singlet-Fission in Carotenoids
Source: J Phys Chem Lett. 2022 Feb 2;13(5):1344–9. doi: 10.1021/acs.jpclett.1c03812 (PMC9084603; doi:10.1021/acs.jpclett.1c03812)
Supplement: Supplementary file 2 — jz1c03812_si_002.pdf [file jz1c03812_si_002.pdf]

jz-2021-038122.R1

Name: Peer Review Information for "Singlet Triplet-Pair Production and Possible Singlet-Fission in Carotenoids"

First Round of Reviewer Comments

Reviewer: 1

Comments to the Author
The article written by Manawadu et al. reports an excellent theoretical work that can help to disentangle the puzzle of the complicated electronic relaxation process from the singlet excited states of carotenoid, which is an urgent issue to be clarified in order to establish the correct understandings of the primary process of photosynthesis that should be applied to realize artificial photosynthesis to produce solar fuels. This paper is successful to explain the possible internal conversion process from the S_2 state of a carotenoid zeaxanthin based on the numerical simulations using time-dependent DMRG method with the extended Hubbard model Hamiltonian, in which U (Coulombic interaction of two electrons in the same orbital) and V (the nearest-neighbor Coulombic repulsion) are parametrized so as to include the long-range Coulombic terms of the Pariser-Parr-Pople-Peierls (PPPP) model of the π-conjugated system. The most significant finding of this work is that the internal conversion from the photoexcited S_2 state of carotenoids to a singlet triplet-pair state occurs adiabatically via an avoided crossing within 100 fs. This mechanism can be good help to interpret the recently found very fast triplet formation in the bacterial light-harvesting system by the group of Hashimoto et al. (see C. Uragami, et al., *J. Photochem. Photobiol. A: Chemistry* **400** (2020) 112628, DOI: 10.1016/j.jphotochem.2020.112628).

The manuscript is basically well written with well understandable form. Therefore, I would like to recommend the publication of this work with its current form. However, in order to improve the quality of this manuscript, I would like to suggest adding the following issues before considering the publication.

(1) In the introduction part, the following nicely written review papers which explain the singlet excited state properties of carotenoids should be included for the purpose that the general readers can more easily catch up the importance of this work.

- T. Polívka and V. Sundström, **Ultrafast dynamics of carotenoids excited states - From solution to natural and artificial systems**, *Chem. Rev.* **104** (2004) 2021-2071, DOI: 10.1021/cr020674n.
- T. Polívka and V. Sundström, **Dark excited states of carotenoids: Consensus and controversy,** *Chem. Phys. Letters* **477** (2009) 1-11, DOI: 10.1016/j.cplett.2009.06.011.
- H. Hashimoto, C. Uragami, N. Yukihira, A. T. Gardiner and R. J. Cogdell, **Understanding/unravelling carotenoid excited singlet states**, *J. R. Soc. Interface* **15** (2018) 20180026, DOI: 10.1098/rsif.2018.0026.

(2) The authors thoroughly try to interpret the singlet excited states of the carotenoid based on the symmetry rule derived from the Hubbard Hamiltonian. However, there is a recent review paper that claims this is not the case (see Fiedor et al., J. R. Soc. Interface **16** (2019) 20190191, DOI: 10.1098/rsif.2019.0191), although I personally do not agree with this thought. Therefore, I want to ask the authors to explain more explicitly how important to interpret the singlet excited states of the carotenoid based

on the symmetry rule. I think that the present study is clearly demonstrating that the inclusion of the $\hat{H}_{SB}$ term might be good enough to explain the factor caused by the symmetry breaking. Please clarify this point for the purpose to save the researchers in this field from the unnecessary confusion from the purely theoretical point of view. Then, the significance of this work will become much more strengthen.

(3) In Figure 2, excitation energies of the diabatic states and adiabatic states show oscillations as a function of time. Is this also caused by the C=C and C-C stretching as can be seen in Figure 3? Please add the explanation.

(4) In Page 8 second line from bottom, abruptly the wording of "the triplet-pair state, $|1\ {}^1B_u^-\rangle$" has appeared. I would like to ask the authors to add explanation how they can assign the $|1\ {}^1B_u^-\rangle$ as the triplet-pair state. I think to explain this with well understandable way is very important, since this assignment is closely related to the final conclusion.

(5) In Page 7 four lines from the bottom, the authors claim "higher energy ${}^1B_u$ states (e.g., $|\Psi_4\rangle$) are also excited by the dipole operator acting on the ground state. Please explain why they can say so. This looks not obvious for me, since $\langle 1\ {}^1B_u^-|\hat{\mu}|1\ {}^1A_g^-\rangle \sim \langle -|\hat{\mu}|-\rangle$ should be considered to be zero.

(6) In Page 11 top, the authors noted that singlet fission from $|2\ {}^1A_g^-\rangle$ is expected to be endothermic for both intra and intermolecular processes. Please add more explanation why they can claim like this way and also explain how large the energy is expected to be necessary to overcome this endothermic process.

(7) Typos

- Abstract, second line from the bottom: … ~50%. We … (a period is missing)
- Page 6, second line from the bottom: which in turn (is should be revised to in).

Reviewer: 2

Comments to the Author

This paper presents a detailed simulation of the internal conversion of the optically allowed (nominally 11Bu+/S2) state to the |TT> state (represented by a nominal 1^1Bu^- in the carotenoid zeaxanthin. They a very rigorous DMRG approach for the electronic degrees of freedom and a classical approach to the nuclear degrees of freedom. This ignores nuclear fluctuations caused by the bath (apart from a phenomenological damping term) but this is quite reasonable given that coherent high frequency nuclear vibrational have been shown to be very important for the S2-S1 relaxation step. It is a very interesting and theoretical work and although it comes from and is pitched to those interested in carotenoid singlet fission it should be of interest to those studying carotenoid excited state relaxation more generally (including its role in biological processes).

My biggest criticism is that, since the authors have decided to study a particular xanthophyll (zeaxanthin), they should at mention how their results compare with the quite large catalogue of spectroscopic data that is out there. I am not suggesting that they calculate optical responses but some simple and brief discussion would make this much more interesting and impactful. For example the <100 fs interconversion seems to align with the ~80 fs obtained from the rise of the induced S_1-S_n absorption in transient absorption measurements on various carotene and

xanthophyll (Polivka, Kennis, Hauer, etc). You mention that the probabilities oscillate at approximately the C-C frequency. How does this relate to the fast oscillations seen in the coherent four wave mixing (particularly the transient grating part) during the S2-S1 decay? Hashimoto's group showed (for beta-carotene and longer homologues) that reflect coherent oscillation in the symmetric C-C, C=C and out-of plane CH_3 modes. Similar measurements by Schlau-Cohen have been performed on xanthophylls. Of particular relevance might by the work on zeaxanthin and longer homologues (by a few people) as this may be relevant to the dependence on conjugation length that you discuss. Purely theoretical papers are important but given how nice this simulation is its impact would be much increased by some discussion.

Minor points
-I think it would be useful to very briefly connect the (a)diabatic states computed in these models to the S_1, S_2, etc levels (strictly should be termed 'signals') that appear in optical measurements. For example, is the spectroscopic S_1 a band of states? Are the 1^1Bu^- and/or 3^1Ag^- related to S_X? It might help people from outside picture the level scheme a bit better and wouldn't need much.
-Why was zeaxanthin chosen? Is it due to its symmetry? Applicability to singlet fission measurements?
-I found the use of the term H_{SB} a bit confusing. Is it a system-bath interaction? My reading is that it is there to account for the fact that bond length alternation varies between the ends of the chain and the centre. It might be worth (in the SI) just clarifying this physically. Saying that it is to reproduce DFT atomic charges is a bit less clear.
-I found the discussion of the dependence of the energy ordering of various vertical and relaxed states hard to visualize without looking up the Koyama paper you referenced. I hadn't yet seen Fig. 1 of the SI so perhaps you could sign-post this a bit better in the main article.
-The table of contents figure seems a bit of an afterthought.

Reviewer: 3

Comments to the Author
The paper is devoted to modeling of the electron-nuclear dynamics in a carotenoid zeaxanthin. Authors use a combination of DMRG for electron dynamics and Ehrenfest equation for nuclear dynamics, a potentially very powerful set of methods. However, in order to apply these methods to a rather large molecule with a complex manifold of electronic states, the authors use phenomenological expressions for electronic and nuclear Hamiltonians which are based on highly debatable assumptions and use a large number of fitting parameters. This fact cast doubts on both the adequacy and the predictive capabilities of the model used. To make the results of the modeling suitable for publication, the authors should address several issues which are listed below.

1. Throughout the paper the authors use the terms "triplet pair" and "singlet fission" to describe the phenomena occurring in the polyene system. Such terminology can be confidently applied either to systems consisting of several weakly coupled moieties or to large polymer molecules. Whether a rather short 11-double bond conjugate system of zeaxanthin is able to contain two triplet solitons as distinct quasiparticles, is not obvious and requires a commentary.

2. The nonadiabatic dynamics between only two states, 1Bu+ and 1Bu-, is explicitly included in the model while the low-lying states of Ag symmetry are only commented on. This is surprising considering that fast 1Bu+ -> 2Ag- transition in polyene systems happens within a similar time period (~100 fs) and is one of their key features. Thus, any attempt to compare the computational results with experiment or make any predictive statements would unavoidably require the inclusion of 2Ag- in the model. The authors make a one-sentence commentary (p.11,

last sentence of the main text) about future expansion of the model, but a more extensive discussion of this is needed.

3. Throughout the paper the zeaxanthin molecule is assumed to be of C2h symmetry and the corresponding notation for state symmetries (Ag, Bu) is used. However, the 1st and 11th double bonds of the molecule are not in the same plane as the other 9 due to the repulsion between the CH3 groups of the rings and the H atoms of the chain. Because of that, the molecule belongs either to Ci or C2 symmetry group, depending on conformation. This non-planarity not only lowers the symmetry of states but also lowers the effective length of the conjugated bond system (see comment #1) and affects the form the nuclear Hamiltonian should take (see comment #7).

4. In discussion of the structure of triplet-pair states (last paragraph on p.3 and further) authors refer to the 4-electron model (refs 12-15) which produces triplet pairs as a combination singlets, triplets and quintuplets. From these references only the 14th is related to polyenes (albeit of a larger size) while others are devoted to polymers or polyacene dimers and similar objects. The authors make no comments about how this picture compares to a broader field of quantum chemical calculations made for polyene systems. For instance, ab initio DMRG and CASSCF calculations show that (4,4) active space is insufficient for describing several main features of 2Ag- and 1Bu- states of polyenes and the minimal appropriate space should be (6,6) [Khokhlov-2020]. In addition, the paper would become more illustrative if the authors could represent the structure of relevant electronic states not only within the site picture pertinent to DMRG but also within the orbital-determinant picture pertinent to MCSCF and other post-Hartree-Fock methods.

5. It is known [Taffet-2017] that different electronic state wavefunctions contain different shares of static and dynamic correlation. The "-" states contain less of the dynamics kind while the "+" states contain more. Considering this fact, the electronic Hamiltonian used in the paper (which accounts only for static correlation) seems not exactly fitting for modeling the 1Bu- <-> 1Bu+ dynamics.

6. The parametrization for the electronic Hamiltonian used in the paper is based on refs 16 and 17. While the latter is related to polyene systems (although not zeaxanthin), the former is dedicated to poly(para-phenylenevinylene) which is a completely different molecular system. At the same time, the authors again ignore a large field of quantum chemical calculations for polyene systems. The E-1/N plots (fig.1 in supplementary and fig.2,3 in ref.14) can not be the only argument for using one or another set or parameters since similar plots for polyenes can be obtained using a very large number of various approaches including as simple ones as the Huckel model.

7. The authors use a one-dimensional nuclear Hamiltonian with displacements of atoms from their equilibrium positions as variables. Such a model would be more fitting for polyynes rather than polyenes. Not only can the relevant vibrational modes be two-dimensional, the out-of-plane twisting movements around C-C bonds can also happen and are known to result in non-adiabatic transitions in polyenes. This phenomenon can not be captured by the model at all. Also, as was already mentioned, the conjugated system in zeaxanthin is itself non-planar.

8. It is implied in the utilized electronic and nuclear Hamiltonians that all C-C bonds in the conjugated system have the same length (related to parameter V) and force constant (parameter K). However, polyenes have very distinct bond length alternation (BLA) patterns specific to certain electronic states. The possible influence of BLA on dynamic behavior should be discussed by the authors.

9. The ultimate results of the modeling reported in the paper are essentially the population-time plots (figs. 3 and 4) which can be characterized by oscillation period, oscillation amplitude, and

mean value. To produce those, the authors use a 7-parameter model (alpha,beta,gamma,omega0,K,U,V). It would be beneficial to discuss the impact of different parameters on the final result and the possibility to obtain the same result with different set of parameters.

10. The authors make no comments about the time-dependent molecular properties related to electron-nuclear dynamics. The modeling of those could be useful in connection to existing or potential spectroscopic studies of polyene molecules.

11. There is a number of typos in the text. For instance, psi3 should be replaced by c3 in eq.5, "optically excited" should be probably replaced by "optically allowed" in line 6 of p.3, etc.

[Khokhlov-2020] J. Phys. Chem. A2020, 124, 5790−5803
[Taffet-2017] Chem. Phys. 515 (2018) 757–767

Author's Response to Peer Review Comments:

Reviewer No. 1

0. Referee: The article written by Manawadu et al. reports an excellent theoretical work that can help to disentangle the puzzle of the complicated electronic relaxation process from the singlet excited states of carotenoid, which is an urgent issue to be clarified in order to establish the correct understandings of the primary process of photosynthesis that should be applied to realize artificial photosynthesis to produce solar fuels. This paper is successful to explain the possible internal conversion process from the S_2 state of a carotenoid zeaxanthin based on the numerical simulations using time-dependent DMRG method with the extended Hubbard model Hamiltonian, in which U (Coulombic interaction of two electrons in the same orbital) and V (the nearest-neighbor Coulombic repulsion) are parametrized so as to include the long-range Coulombic terms of the Pariser-Parr-Pople-Peierls (PPPP) model of the p-conjugated system. The most significant finding of this work is that the internal conversion from the photoexcited S_2 state of carotenoids to a singlet triplet-pair state occurs adiabatically via an avoided crossing within 100 fs. This mechanism can be good help to interpret the recently found very fast triplet formation in the bacterial light-harvesting system by the group of Hashimoto et al. (see C. Uragami, et al., J. Photochem. Photobiol. A: Chemistry 400 (2020)).

The manuscript is basically well written with well understandable form. Therefore, I would like to recommend the publication of this work with its current form. However, in order to improve the quality of this manuscript, I would like to suggest adding the following issues before considering the publication.

0. Reply: We thank the referee for spending their time reviewing our paper and for their constructive criticisms of it, which we address below.

1. Referee: In the introduction part, the following nicely written review papers which explain the singlet excited state properties of carotenoids should be included for the purpose that the general readers can more easily catch up the importance of this work.
T. Polívka and V. Sundström, Chem. Rev. 104 (2004) 2021-2071.
T. Polívka and V. Sundström, Chem. Phys. Letters 477 (2009).
H. Hashimoto, C. Uragami, N. Yukihira, A. T. Gardiner and R. J. Cogdell,, J. R. Soc. Interface 15 (2018) 20180026.
Reply and changes: We thank the referee for the suggestion of these very useful references. The 1st and 3rd are now referenced in the introduction (see p.6).

2. Referee: The authors thoroughly try to interpret the singlet excited states of the carotenoid based on the symmetry rule derived from the Hubbard Hamiltonian. However, there is a recent review paper that claims this is not the case (see Fiedor et al., J. R. Soc. Interface 16 (2019) 20190191, although I personally do not agree with this thought. Therefore, I want to ask the authors to explain more explicitly how important to interpret the singlet excited states of the carotenoid based on the

symmetry rule. I think that the present study is clearly demonstrating that the inclusion of the H_{SB} term might be good enough to explain the factor caused by the symmetry breaking. Please clarify this point for the purpose to save the researchers in this field from the unnecessary confusion from the purely theoretical point of view. Then, the significance of this work will become much more strengthen.

Reply: We agree with [Fiedor-2019] that the absence of the S_0 -> S_1 transition does not arise from selection rules due to spatial-symmetry, but is due to the inherent electronic nature of S_1 (and indeed of the "$2A_g$" family of singlet triplet-pair states), namely their triplet-triplet character and excitonic character with odd electron-hole parity.

Changes: None, as this discussion seems to be beyond the scope of the current paper. (Besides, this point seems to have been answered by the referee themselves in their question #5 below.)

3. Referee: In [old] Figure 2, excitation energies of the diabatic states and adiabatic states show oscillations as a function of time. Is this also caused by the C=C and C-C stretching as can be seen in [old] Figure 3? Please add the explanation.

Reply: We think that the oscillations shown in (new) Fig. 3, as in (new) Fig. 5, are associated with C-C bond stretches (however, the oscillations shown in (new) Fig. 4 are non-stationary state oscillations).

Changes: We have mentioned this point in the caption to (new) Fig. 5.

4. Referee: In Page 8 second line from bottom, abruptly the wording of "the triplet-pair state, $|1\ ^1B_u^-\rangle$" appeared. I would like to ask the authors to add explanation how they can assign the $|1\ ^1B_u^-\rangle$ as the triplet-pair state. I think to explain this with well understandable way is very important, since this assignment is closely related to the final conclusion.

Reply: In earlier work on longer polyene chains [Valentine-2020], using energetic considerations, bond-dimerization, spin-spin correlation and triplet-pair overlaps (for 12 C-atoms), we showed that the "$2A_g$ family" (i.e., $2\ ^1A_g^-$, $1\ ^1B_u^-$, $3\ ^1A_g^-$ ....) have strong triplet-triplet pair character. In this work we have assumed that this character is present for these states on shorter chains. However, we agree with the referee that this should be better established for carotenoids, so we have made the changes below.

Changes: We have included a section in the SI showing the bond-dimerization (and hence the four-soliton structure) of $2\ ^1A_g^-$ and $1\ ^1B_u^-$ states, which is quantitatively the same as for longer chains. (See Sec. 4 of the SI and the note at the top of p.11.)

5. Referee: In Page 7 four lines from the bottom, the authors claim "higher energy $1\ ^1B_u^-$ states (e.g., $|\psi_4\rangle$) are also excited by the dipole operator acting on the ground state". Please explain why they can say so. This looks not obvious for me, since $\langle 1\ ^1B_u^- |\mu| 1\ ^1A_g^- \rangle$ should be considered to be zero.

Reply: $|\psi_4\rangle$ (in the old notation) has a small probability (less than 1%) of being excited from the ground state by the dipole operator because the Hamiltonian does not possess particle-hole symmetry, and thus there are no selection rules to prevent it (see #2 above). However, most of the

additional oscillator strength comes from (nominal) $2\ ^1B_u^+$, $3\ ^1B_u^+$ …. states, so our statement was indeed misleading.
Changes: In the new version we take as our initial condition $\Psi(t=0) = |S_2\rangle$ (or $|\psi_3\rangle$ in the old notation), as this simplifies the two-state analysis. Thus, this point no longer arises and it is not discussed.

6. Referee: In Page 11 top, the authors noted that singlet fission from $|2\ ^1A_g^-\rangle$ is expected to be endothermic for both intra and intermolecular processes. Please add more explanation why they can claim like this way and also explain how large the energy is expected to be necessary to overcome this endothermic process.
Reply: As shown in [Valentine-2020], the quintet corresponds to two unbound triplets on the same chain. Thus, the quintet-$2A_g$ (relaxed) positive energy gap is the energy to unbind the triplets in the $2A_g$ state on the same chain. This is 0.4 eV for typical carotenoid chain lengths. Similarly, the $2xT_1$-$2A_g$ energy gap corresponds the energy to unbind the triplets in the $2A_g$ state on two separate chains of the same length. This is 0.6 eV for typical carotenoid chain lengths.
Changes: We have made this discussion more explicit (see middle p.13).

7. Referee: Typos
Abstract, second line from the bottom: …
50%. We … (a period is missing)
Page 6, second line from the bottom: which in turn (is should be revised to in).
Reply and changes: Agreed and corrected.

[Fiedor-2019] Fiedor et al., J. R. Soc. Interface 16 (2019) 20190191
[Valentine-2020] Valentine, D. J.; Manawadu, D.; Barford, W, Physical Review B 2020, 102.

Reviewer No. 2

0. Referee: This paper presents a detailed simulation of the internal conversion of the optically allowed (nominally $1\ ^1B_u^+/S_2$) state to the |TT> state (represented by a nominal $1\ ^1B_u^-$ in the carotenoid zeaxanthin. They [use] a very rigorous DMRG approach for the electronic degrees of freedom and a classical approach to the nuclear degrees of freedom. This ignores nuclear fluctuations caused by the bath (apart from a phenomenological damping term) but this is quite reasonable given that coherent high frequency nuclear vibrational have been shown to be very important for the S_2-S_1 relaxation step. It is a very interesting and theoretical work and although it comes from and is pitched to those interested in carotenoid singlet fission it should be of interest to those studying carotenoid excited state relaxation more generally (including its role in biological processes).
0. Reply: We thank the referee for spending their time reviewing our paper and for their constructive criticisms of it, which we address below.

1. Referee: My biggest criticism is that, since the authors have decided to study a particular xanthophyll (zeaxanthin), they should at mention how their results compare with the quite large catalogue of spectroscopic data that is out there. I am not suggesting that they calculate optical responses but some simple and brief discussion would make this much more interesting and impactful. For example the <100 fs interconversion seems to align with the ~80 fs obtained from the rise of the induced S_1-S_n absorption in transient absorption measurements on various carotene and xanthophyll (Polivka, Kennis, Hauer, etc).
Reply: Although we did reference some spectroscopic data (and have added more), in this short letter we have focused on the dynamics of the internal conversion. Our intention is to calculate the transient absorption in the near future. We will then submit a regular article with these results and comparisons to the spectroscopic data.
Changes: We have, however, made additional references to spectroscopic data and discussed the predicted photoexcited absorption (see p. 13).

2. Referee: You mention that the probabilities oscillate at approximately the C-C frequency. How does this relate to the fast oscillations seen in the coherent four wave mixing (particularly the transient grating part) during the S_2-S_1 decay? Hashimoto's group showed (for beta-carotene and longer homologues) that reflect coherent oscillation in the symmetric C-C, C=C and out-of plane CH_3 modes. Similar measurements by Schlau-Cohen have been performed on xanthophylls. Of particular relevance might by the work on zeaxanthin and longer homologues (by a few people) as this may be relevant to the dependence on conjugation length that you discuss. Purely theoretical papers are important but given how nice this simulation is its impact would be much increased by some discussion.
Reply: We thank the referee for these observations. These are important questions and we agree that it is necessary to interpret experimental results. However, without further work we cannot directly address them. As mentioned above, it is our intention to do so in the near future.

Changes: None.

3. Referee: I think it would be useful to very briefly connect the (a)diabatic states computed in these models to the S_1, S_2, etc levels (strictly should be termed 'signals') that appear in optical measurements. For example, is the spectroscopic S_1 a band of states? Are the $1\ ^1B_u^-$ and/or $3\ ^1A_g^-$ related to S_X? It might help people from outside picture the level scheme a bit better and wouldn't need much.
Reply and changes: We have changed our notation to make explicit that our adiabatic states are S_2 and S_3 (S_1 is $2A_g$). We think that this new notation and (new) Fig. 3 obviates the need for an energy-level diagram. At present we are unable to answer the question whether $1\ ^1B_u^-$ and/or $3\ ^1A_g^-$ are related to S_X; we hope that our planned transient absorption calculations will address it.

4. Referee: Why was zeaxanthin chosen? Is it due to its symmetry? Applicability to singlet fission measurements?
Reply and changes: It was chosen partly because it has a higher symmetry than other carotenoids, thus making the internal conversion easier to interpret. It was also chosen because of its applicability to singlet fission measurements, so a reference has been added for that point [Musser15].

5. Referee: I found the use of the term H_{SB} a bit confusing. Is it a system-bath interaction? My reading is that it is there to account for the fact that bond length alternation varies between the ends of the chain and the centre. It might be worth (in the SI) just clarifying this physically. Saying that it is to reproduce DFT atomic charges is a bit less clear.
Reply: The term H_{SB} (now labelled H_ε to avoid misunderstandings) is a symmetry-breaking term introduced to mix the $1\ ^1B_u^+$ and $1\ ^1B_u^-$ states.
Changes: We have made this more explicit in the main text (see the text on p.7 preceding eqn (5)).

6. Referee: I found the discussion of the dependence of the energy ordering of various vertical and relaxed states hard to visualize without looking up the Koyama paper you referenced. I hadn't yet seen Fig. 1 of the SI so perhaps you could sign-post this a bit better in the main article.
Reply and changes: We have now moved this figure into the main paper (new Fig. 1).

7. Referee: The table of contents figure seems a bit of an afterthought.
Reply and changes: We have submitted a new version.

[Musser15] A. J. Musser, et al. JACS, 2015, **137**, 5130–5139.

Reviewer No. 3

0. Referee: The paper is devoted to modeling of the electron-nuclear dynamics in a carotenoid zeaxanthin. [The] authors use a combination of DMRG for electron dynamics and Ehrenfest equation for nuclear dynamics, a potentially very powerful set of methods. However, in order to apply these methods to a rather large molecule with a complex manifold of electronic states, the authors use phenomenological expressions for electronic and nuclear Hamiltonians which are based on highly debatable assumptions and use a large number of fitting parameters. This fact cast doubts on both the adequacy and the predictive capabilities of the model used. To make the results of the modeling suitable for publication, the authors should address several issues which are listed below.

0. Reply: We thank the referee for spending their time reviewing our paper and for their criticisms of it. Some of these criticisms are constructive and help improve our paper (i.e., #1, #2, #3). Others however, given the scope of our calculations, seem to us to be unreasonable (i.e., #4, #5, #6). We address them below.

The referee seems to recognise that we have applied a powerful set of computational methods to this very difficult computational problem, without also recognising that this would be impossible using a fully *ab initio* Hamiltonian. Our model contains the key ingredients required to describe the highly-correlated states of carotenoids and their coupling to nuclear degrees of freedom. Furthermore, we have only two-fitting parameters, namely U and V, which were fitted to our previous Pariser-Parr-Pople-Peierls calculations.

1. Referee: Throughout the paper the authors use the terms "triplet pair" and "singlet fission" to describe the phenomena occurring in the polyene system. Such terminology can be confidently applied either to systems consisting of several weakly coupled moieties or to large polymer molecules. Whether a rather short 11-double bond conjugate system of zeaxanthin is able to contain two triplet solitons as distinct quasiparticles, is not obvious and requires a commentary.
Reply: In earlier work on longer polyene chains [Valentine-2020], using energetic considerations, bond-dimerization, spin-spin correlation and triplet-pair overlaps (for 12 C-atoms), we showed that the “2A_g family” (i.e., 2 $^1A_g^-$, 1 $^1B_u^-$, 3 $^1A_g^-$ ....) have strong triplet-triplet pair character. In this work we have assumed that this character is present for these states on shorter chains. However, we agree with the referee that this should be better established for carotenoids, so we have made the changes below.
Changes: We have included a section in the SI showing the bond-dimerization (and hence the four-soliton structure) of 2 $^1A_g^-$ and 1 $^1B_u^-$ states, which is quantitatively the same as for longer chains. (See Sec. 4 of the SI.)

2. Referee: The nonadiabatic dynamics between only two states, $1B_u^+$ and $1B_u^-$, is explicitly included in the model while the low-lying states of A_g symmetry are only commented on. This is surprising considering that fast B_u^+ -> A_g^- , transition in polyene systems happens within a similar time period

(~100 fs) and is one of their key features. Thus, any attempt to compare the computational results with experiment or make any predictive statements would unavoidably require the inclusion of $2A_g^-$ in the model. The authors make a one-sentence commentary (p.11, last sentence of the main text) about future expansion of the model, but a more extensive discussion of this is needed.
Reply: The purpose of this work is to establish internal conversion from S_2 to the singlet triplet-pair manifold. We chose a high-symmetry molecule where only $1B_u^+$ to $1B_u^-$ internal conversion is expected. We stated that we do intend to study more general systems where $1B_u^+$ to $2A_g^-$ internal conversion occurs.
Changes: We expand on this point briefly in the conclusions (see p. 14).

3. Referee: Throughout the paper the zeaxanthin molecule is assumed to be of C_{2h} symmetry and the corresponding notation for state symmetries (A_g, B_u) is used. However, the 1st and 11th double bonds of the molecule are not in the same plane as the other 9 due to the repulsion between the CH_3 groups of the rings and the H atoms of the chain. Because of that, the molecule belongs either to C_i or C_2 symmetry group, depending on conformation. This non-planarity not only lowers the symmetry of states but also lowers the effective length of the conjugated bond system (see comment #1) and affects the form the nuclear Hamiltonian should take (see comment #7).
Reply: We thank the referee for this point and agree with them. Consequently, we have added a footnote (#4 p.8) about the symmetry. More importantly, we have modified the electron transfer integral on the 2nd and 20th bond to $\beta \cos \phi$ with the dihedral angle $\phi = 75^0$ (from literature values [Bartalucci-2007]). This does indeed reduce the effective conjugation length to 9 double bonds, thus changing the excitation energies (see Fig. 1). However, it does not change the relative energy orderings and does not qualitatively change our predictions.
Changes: See Sec. 1 of the SI and our new internal conversion results in (new) Figs 3-5.

4(a) Referee: In [the] discussion of the structure of triplet-pair states (last paragraph on p.3 and further) [the] authors refer to the 4-electron model (refs 12-15) which produces triplet pairs as a combination singlets, triplets and quintuplets. From these references only the 14th is related to polyenes (albeit of a larger size) while others are devoted to polymers or polyacene dimers and similar objects.
Reply: The coupling of two particles of spin 1, i.e., the combination of two triplets: TxT = S + T + Q, can be determined from the Clebsch-Gordon coefficients of angular momentum. This is independent of the 4-electron model, so we are unclear what the referee means by this point. We mainly added these references to put our work in context of other singlet-fission systems.
Changes: None.

4(b) Referee: The authors make no comments about how this picture compares to a broader field of quantum chemical calculations made for polyene systems. For instance, ab initio DMRG and CASSCF calculations show that (4,4) active space is insufficient for describing several main features of $2A_g^-$ and $1B_u^-$ states of polyenes and the minimal appropriate space should be (6,6) [Khokhlov-2020]. In addition, the paper would become more illustrative if the authors could represent the

structure of relevant electronic states not only within the site picture pertinent to DMRG but also within the orbital-determinant picture pertinent to MCSCF and other post-Hartree-Fock methods.

Reply: The reason why DMRG is so successful for one-dimensional quantum lattice Hamiltonians is explained by the underlying theory of Matrix Product States (MPS). This theory states that when the interactions are short-ranged, an "area law" is obeyed, meaning that DMRG converges to a specified accuracy for a finite-size Hilbert space. As our Hamiltonian is a one-dimensional lattice Hamiltonian with short-range interactions, it explicitly satisfies the criteria for the area law, and thus as our calculations are converged we rigorously describe all electronic correlation (both static and dynamic) within our single-particle basis. DMRG post-Hartree-Fock *ab initio* methods, in contrast, can be unreliable because they convert short-range interactions into long-range four-centre integrals, thus violating the "area law".

Changes: In this short letter we do not wish to review all electronic structure calculations on carotenoids. However, as [Khokhlov-2020] is related to our work, this and two others [Hu-2015, Taffet-2019], are now referenced (see p.6).

5. Referee: It is known [Taffet-2018] that different electronic state wavefunctions contain different shares of static and dynamic correlation. The "-" states contain less of the dynamics kind while the "+" states contain more. Considering this fact, the electronic Hamiltonian used in the paper (which accounts only for static correlation) seems not exactly fitting for modeling the $1B_u^-$ <-> $1B_u^+$ dynamics.
Reply: As stated above (#4(b)) DMRG accurately computes all the static and dynamic correlation within its single-particle basis. As this single-particle basis is truncated, however, it is possible that some dynamic correlation (by which we think the referee means polarization) is explicitly neglected. This can often be implicitly remedied by suitably parametrizing reduced-basis Hamiltonians [Castleton-2002]. We have found that a small reduction in the Hubbard U leads to reversal of the vertical $1B_u^+$ and $2A_g^-$ energies, whose consequence we will investigate shortly.
Changes: None.

6. Referee: The parametrization for the electronic Hamiltonian used in the paper is based on refs 16 and 17. While the latter is related to polyene systems (although not zeaxanthin), the former is dedicated to poly(para-phenylenevinylene) which is a completely different molecular system. At the same time, the authors again ignore a large field of quantum chemical calculations for polyene systems. The E-1/N plots (fig.1 in supplementary and fig.2,3 in ref.14) cannot be the only argument for using one or another set or parameters since similar plots for polyenes can be obtained using a very large number of various approaches including as simple ones as the Huckel model.

Reply: We do not understand the referee's point. Yes, poly(para-phenylenevinylene) and zeaxanthin have different molecular structures, but they are both π-conjugated carbon molecules. Why should there necessarily be a different set of parameters for these two systems? Indeed, because of their

different structures, solving the PPP model with the same parameters for these two systems gives very different electronic predictions [Barford-2013]. In addition, as stated in our paper, our calculated energies for carotenoids are in good agreement with experiment.

The referee's comment "similar plots for polyenes can be obtained using a very large number of various approaches including as simple ones as the Huckel model" is simply incorrect. A non-interacting (Huckel) model of polyenes cannot predict a $1\ ^1B_u^+/2\ ^1A_g^-$ energy level reversal, because of the nodal structure of the one-electron wavefunctions. Electronic correlations are necessary to account for this reversal (see (#5) above) [Barford-2013].

Nonetheless, we do concede that the relative vertical energies of the $1\ ^1B_u^+$ and $2\ ^1A_g^-$ states is controversial, with high-level *ab initio* DMRG quantum chemistry calculations placing $2\ ^1A_g^-$ above $1\ ^1B_u^+$ [Taffet-2018, Khokhlov-2020]. As stated above (#5) future work will investigate that scenario.

Changes: We have added references to recent quantum chemical calculations for polyene systems (see #4(b)).

7. Referee: The authors use a one-dimensional nuclear Hamiltonian with displacements of atoms from their equilibrium positions as variables. Such a model would be more fitting for polyynes rather than polyenes. Not only can the relevant vibrational modes be two-dimensional, the out-of-plane twisting movements around C-C bonds can also happen and are known to result in non-adiabatic transitions in polyenes. This phenomenon cannot be captured by the model at all. Also, as was already mentioned, the conjugated system in zeaxanthin is itself non-planar.
Reply: Although out-of-plane twisting might cause non-adiabatic transitions, the time scales for these will be much longer than the 10s of fs timescales considered here. While it would be interesting to include these in further work to understand the longer-time dynamics (e.g., for singlet fission), we do not expect them to be important for ultrafast internal conversion. The non-planarity of zeaxanthin is addressed in (see #3).
Changes: None.

8. Referee: It is implied in the utilized electronic and nuclear Hamiltonians that all C-C bonds in the conjugated system have the same length (related to parameter V) and force constant (parameter K). However, polyenes have very distinct bond length alternation (BLA) patterns specific to certain electronic states. The possible influence of BLA on dynamic behavior should be discussed by the authors.
Reply: We have found in previous work [Barford-2001] that corrections arising from the changes in the nearest-neighbor repulsion arising from changes in bond lengths are negligible. However, we will comment on this point. K will not change for harmonic springs. (The bond integral, β, does change during the dynamics.)
Changes: See footnote #2 on p.7.

9. Referee: The ultimate results of the modeling reported in the paper are essentially the population-time plots (figs. 3 and 4) which can be characterized by oscillation period, oscillation amplitude, and mean value. To produce those, the authors use a 7-parameter model (α, β, γ, ω_0, K, U, V). It would be beneficial to discuss the impact of different parameters on the final result and the possibility to obtain the same result with different set of parameters.
Reply: As stated above, our parameters are relevant for carotenoids. A wider discussion of parameter space lies outside the scope of this paper.
Changes: None.

10. Referee: The authors make no comments about the time-dependent molecular properties related to electron-nuclear dynamics. The modeling of those could be useful in connection to existing or potential spectroscopic studies of polyene molecules.
Reply: Although we did reference some spectroscopic data (and have added more), in this short letter we have focused on the dynamics of the internal conversion. Our intention is to calculate the transient absorption in the near future. We will then submit a regular article with these results and comparisons to the spectroscopic data.
Changes: We have, however, made additional references to spectroscopic data and discussed the predicted photoexcited absorption (see p.13).

11. Referee: There is a number of typos in the text. For instance, psi3 should be replaced by c3 in eq.5, "optically excited" should be probably replaced by "optically allowed" in line 6 of p.3, etc.
Reply: These are not typos, but we have changed our notation to avoid confusion.

[Barford-2001] Barford, W.; Bursill, R. J.; Lavrentiev, M. Y.; Physical Review B 2001, 63
[Barford-2013] Barford, W., *Electronic and Optical Properties of Conjugated Polymers*, 2nd ed.; Oxford University Press: Oxford, 2013.
[Bartalucci-2007] Bartalucci, G.; Coppin, J.; Fisher, S.; Hall, G.; Helliwell, J. R.; Helliwell, M.; Liaaen-Jensen, S. Acta Crystallographica Section B, 2007, **63**, 328.
[Castleton-2002] C. W. M. Castleton and W. Barford, *J. Chem. Phys.*, **117**, 3570 (2002)
[Hu-2015] Hu, W. F.; Chan, G. K. L., J. Chem Theory and Comp. 2015, 11, 3000–3009
[Khokhlov-2020] J. Phys. Chem. A (2020) 124, 5790−5803
[Taffet-2018] Chem. Phys. 515 (2018) 757–767
[Taffet-2019] Taffet, E. J.; Lee, B. G.; Toa, Z. S. D.; Pace, N.; Rumbles, G.; Southall, J.; Cogdell, R. J.; Scholes, G. D., J. Phys. Chem. B 2019, 123, 8628–8643
[Valentine-2020] Valentine, D. J.; Manawadu, D.; Barford, W.; Physical Review B 2020, 102

List of Changes

1. We have modelled the twisted end groups of zeaxanthin by adjusting the electron transfer integrals for the 2nd and 20th C-C bonds. This changes the energy gaps, but there are no changes to any quantitative results.
2. We now take our initial state at time t = 0 to be entirely the primary photoexcited state, S_2, so as to simplify the process of internal conversion. (In our earlier submission, the initial state was $\mu|GS\rangle$, which is 80% composed of S_2.)
3. A new Section 4 in the SI describing bond dimerization and the triplet-triplet character of the $2A_g^-$ and $1B_u^-$ states.
4. To clarify the discussion, we have moved Fig. S1 (from the SI) to the main paper.
5. A change of some notation.
6. Other points of clarification, as recommended by the referees.
